# Path Planning for Unmanned Surface Vehicles with Strong Generalization Ability Based on Improved Proximal Policy Optimization

**DOI:** 10.3390/s23218864

**Published:** 2023-10-31

**Authors:** Pengqi Sun, Chunxi Yang, Xiaojie Zhou, Wenbo Wang

**Affiliations:** 1 Faculty of Mechanical and Electrical Engineering, Kunming University of Science and Technology, Kunming 650500, China; spq5597@stu.kust.edu.cn (P.S.); ycx@kmust.edu.cn (C.Y.); zxj984256@stu.kust.edu.cn (X.Z.); 2 Yunnan International Joint Laboratory of Intelligent Control and Application of Advanced Equipment, Kunming 650500, China

**Keywords:** USV, path planning, deep reinforcement learning, deep neural network, generalization, perception

## Abstract

To solve the problems of path planning and dynamic obstacle avoidance for an unmanned surface vehicle (USV) in a locally observable non-dynamic ocean environment, a visual perception and decision-making method based on deep reinforcement learning is proposed. This method replaces the full connection layer in the Proximal Policy Optimization (PPO) neural network structure with a convolutional neural network (CNN). In this way, the degree of memorization and forgetting of sample information is controlled. Moreover, this method accumulates reward models faster by preferentially learning samples with high reward values. From the USV-centered radar perception input of the local environment, the output of the action is realized through an end-to-end learning model, and the environment perception and decision are formed as a closed loop. Thus, the proposed algorithm has good adaptability in different marine environments. The simulation results show that, compared with the PPO algorithm, Soft Actor–Critic (SAC) algorithm, and Deep Q Network (DQN) algorithm, the proposed algorithm can accelerate the model convergence speed and improve the path planning performances in partly or fully unknown ocean fields.

## 1. Introduction

In recent years, there has been an increasing global emphasis on the importance of marine resources, coinciding with the rapid advancement of artificial intelligence. In this context of technological convergence, Unmanned Surface Vehicles (USVs) have gained considerable traction in various fields including scientific research, ocean resource exploration, water rescue missions, and environmental initiatives [1,2,3,4]. Given the inherently complex and dynamic nature of the marine environment, effective path planning for USVs plays a crucial role in ensuring the successful execution of the aforementioned tasks.

The navigation system of Unmanned Surface Vehicles (USVs) comprises three major subsystems: environmental and navigation situation awareness, cognition-based decision making, and ship navigation control. Path planning and obstacle avoidance are fundamental challenges in constructing these subsystems [5]. A typical path planning task aims to provide collision-free navigation from the starting position to the specified target position on a given map or grid [6,7]. Currently, USV path planning and obstacle avoidance techniques can be broadly classified into traditional methods and intelligent methods. Traditional methods typically refer to deterministic approaches [8] that provide solutions following predefined rules, using fused information at each decision step. Among traditional methods, Iijima et al. [9] used the width-first search method to select and plan collision avoidance paths. However, their approach did not consider the influence of the navigation environment. Churkin et al. [10] attempted to establish a mathematical model for collision avoidance path planning using both continuous and discrete research methods. However, the continuous method exhibited high computational complexity and was unsuitable for cases involving multiple USV encounters. In another study, Hwang et al. [11] employed the fuzzy set theory to establish a knowledge base system to evaluate ship collision risk and determine collision avoidance strategies. However, their system solely focused on collision avoidance strategies, rendering the suboptimal overall voyage. Chang et al. [12] proposed a model for calculating collision avoidance paths on grid maps using a maze routing algorithm, but this approach did not account for international maritime collision avoidance rules and navigation environment conditions.

Szlapcynski et al. [13] improved the maze routing method in [12] by adding a turning penalty and a time-varying restricted area. However, the resulting calculated path remained suboptimal due to the neglect of navigation environment conditions.

Apart from the aforementioned modeled based methods, a number of heuristic algorithms have also been proposed. Recently, a novel Voronoi–Visibility path planning algorithm, which integrates the advantages of a Voronoi diagram and a Visibility graph, was proposed for solving the USV path planning problem in [14]. In [15], Nie et al. studied the problem of robot path planning using the Dijkstra algorithm and Ant Colony Optimization. For known environments, the path planning problem was studied in [16], which introduced geometric areas to divide obstacle avoidance zones and perform global obstacle-avoidance planning of USV with an improved A-star algorithm. In [17], Yao et al. proposed a hierarchical architecture using the biased min-consensus method to address the path planning problem of USVs. In [18], Wu et al. investigated USV path planning by proposing a global path planning approach for USVs using an intelligent water drops algorithm. Wei et al. [19] designed a trajectory planning unit based on the unique characteristics of USVs, reflecting the intelligent navigation of USVs. The aforementioned methods demonstrate a wide application prospect in the field of USVs.

However, determining the optimal obstacle-avoidance path for USVs involves a number of crucial factors, including navigation environmental conditions and international maritime collision-avoidance rules. Many of these factors are abstract and qualitative, making them challenging to quantify using deterministic mathematical methods. In contrast, intelligent methods, such as Deep Reinforcement Learning (DRL) algorithms, show better efficacy in handling abstract and qualitative influencing factors, making them more suitable for USV path planning and obstacle avoidance in uncertain and time-varying ocean environments. DRL combines the feature interpretation capabilities of deep learning with the decision-making abilities of reinforcement learning, enabling direct optimal decision outputs based on high dimensional input data. This approach constitutes an end-to-end decision control system [20,21]. Trained with DRL, Jaradat et al. [22] incorporated a predictive model into DRL, achieving high dynamic performance in convergence speed, average reward value, and other indicators through path planning experiments on aircraft carrier decks. Guan et al. [23] proposed a local path planning and behavior decision-making approach based on an improved Proximal Policy Optimization (PPO) algorithm, enabling smart USVs to reach their targets without requiring human experience. To further enhance ship path planning during navigation, Guo et al. [24] introduced a coastal ship path planning model based on the Deep Q-Network (DQN) algorithm. Prianto et al. [25] developed a path planning algorithm based on Soft Actor–Critic (SAC), allowing for multi-arm manipulator path planning.

Convolutional layers have been widely applied to the feature extraction problem of high-dimensional state tasks in DRL. Habib et al. [26] gave detailed insight into computation acceleration using stochastic gradient descent, fast convolution, and parallelism in CNN. Lebedev et al. [27] covered the approaches based on tensor decompositions, weight quantization, weight pruning, and teacher–student learning. Krichen [28] provided a comprehensive overview of CNNs and their applications in image recognition tasks.

In this paper, a path planning algorithm of USV with local environmental information perception for a time-varying maritime environment is proposed based on an improved Proximal Policy Optimization (PPO) algorithm. The contributions of this study can be summarized in the following three key aspects:

- To reflect realistic maritime environments, a grid-based environment model is constructed based on real-world electronic charts to map the dynamic states of a ship and static obstacles in the sea.
- Integration of planning and obstacle avoidance is achieved based on the proposed PPO algorithm, with the consideration of the sensing range of on-board sensors.
- To address the unpredictable situations, e.g., unknown maps or moving ships in the area, we use convolutional neural networks (CNNs) for state-feature extraction in PPO. Our simulation results show that this method greatly improves the adaptability of USV in path planning in uncharted marine environments.

The rest of this paper is organized as follows. The problem formulation is described in Section 2. In Section 3, a path planning algorithm based on PPO is proposed. Section 4 presents a comparative analysis of the simulation experiment process and experimental results. Finally, the conclusion and future work are given in Section 5.

## 2. Modeling and Problem Formulation

To accurately represent the significant environmental characteristics of the sea, this study adopts the raster model proposed by [29], using electronic nautical charts. The marine environment model includes the raster model, physical model, numerical model, and intelligent model. The raster model has the characteristics of high spatial resolution, data analysis, and visualization. These characteristics enable the model to better capture the detailed features (e.g., coastline and seabed topography) in different areas of the ocean. The output data generated by the grid model can be analyzed and visualized in various forms. Thus, the raster model and electronic nautical charts are leveraged to transform both stationary and dynamic obstacles within a specified sea area into their corresponding raster representations.

### 2.1. Building a Marine Environment Map Based on Electronic Charts

**Figure 1:**
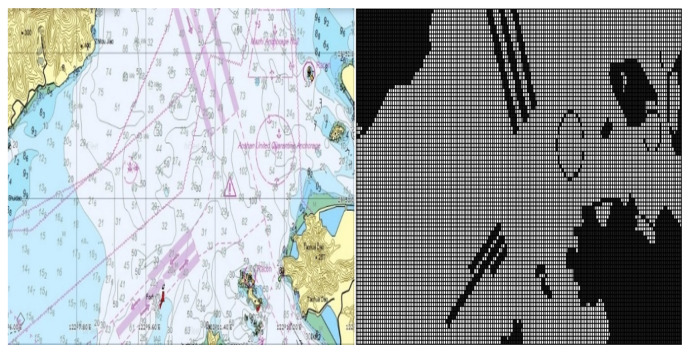
Electronic nautical charts of a given area [4].

The detailed information about the marine environment is extracted from the electronic chart. Firstly, a specific local electronic chart is selected from the global electronic chart, as exemplified in Figure 1. In this example, the chosen chart corresponds to the actual water area of the customized No. 1 warning area within the Ningbo–Zhoushan core port region (regional range: 122.106833${}^{\circ }$ E∼122.286833${}^{\circ }$ E, 29.817667${}^{\circ }$ N∼29.893333${}^{\circ }$ N). Subsequently, grid maps representing the relevant maritime area are generated through several steps, including land information extraction and binary grid processing. Further details regarding the specific grid map relevant to the electronic chart is provided on the right-hand side of Figure 1. Such an approach of grid processing retains the outlines of obstacles (such as lands and islands). Static obstacles such as highways and anchor points can be further added to the grid according to maritime traffic rules. By doing so, reliable information is provided to construct a high-accuracy grid model of the sea environment of interest.

### 2.2. Problem Formulation

**Figure 2:**
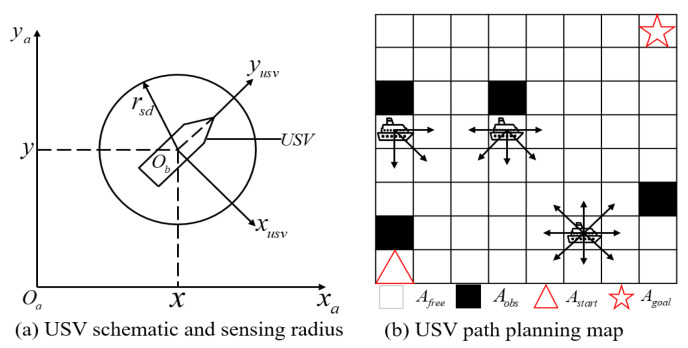
USV schematic and sensing radius. The starting position $A_{start}$ = (1, 1), the goal position $A_{goal}$ = (8, 8), $A_{free}$ is the position that can be passed, and $A_{obs}$ is the obstacle. This paper aims to plan the safe path of USV from the starting position to the goal position.

The diagram that illustrates the USV and the definition of the sensing radius is presented in Figure 2a, where $x_{a}$ and $y_{a}$ represent the coordinates in the earth coordinate system, and $x_{usv}$ and $y_{usv}$ represent the coordinates in the ship coordinate system. In this study, the USV sensing radius is denoted by $r_{sd}$, and the white grid and black grid are defined as $A_{free}$ and $A_{obs}$, respectively. The white grid $A_{free}$ corresponds to the feasible area, while the black grid $A_{obs}$ represents the obstacles such as land, ships, and other obstacles on the sea surface.

In this study, we discretize the action space of a USV into a finite set. As illustrated in Figure 2b, when no obstacles are present around the USV, it is able to move in eight adjacent directions within the grid representation of the sea environment. Additionally, when it is at the edge of the finite grid map, all available movement directions lead towards the interior of the map.

## 3. USV Path Planning Based on PPO

### 3.1. State Space

**Figure 3:**
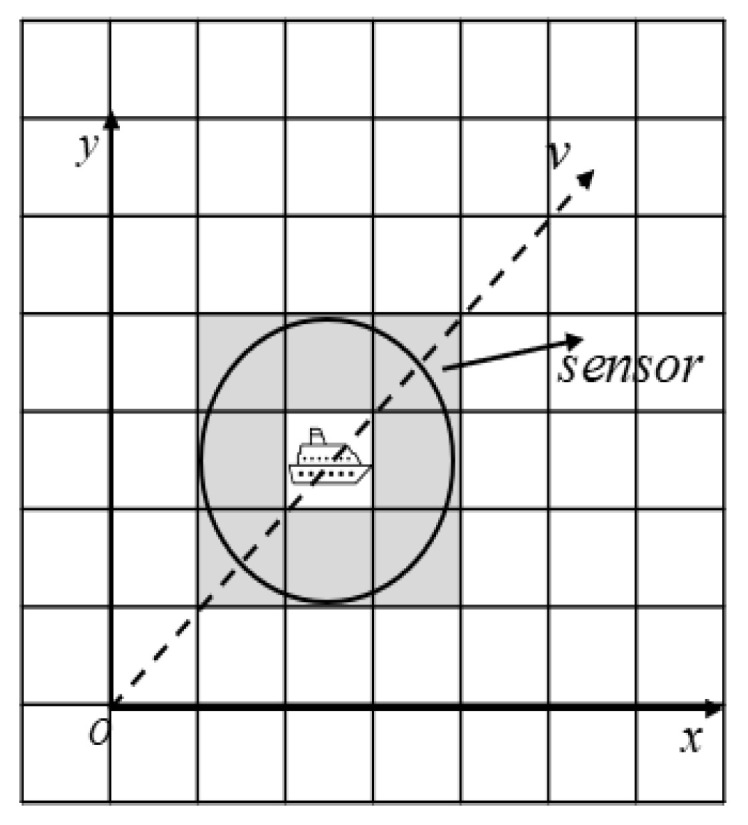
Radar sensor detection range.

To generalize the real-world application scenarios, we assume in this paper that an environment sensor (e.g., an ultra wide-band sensor) is deployed on the USV for sea environment detection. The detection range of the radar sensor is set according to the mapping granularity of the raster environment model. For instance, Figure 3 shows a sensing range of unit length on the grid map as given in Figure 1, which corresponds to the actual length of 102 m at sea. In this paper, we adopt the USV parameters from the literature [20], where a USV performs path planning at a sailing speed of 9 nautical miles per hour. As illustrated in Figure 3, we consider the shaded part in the figure as the detection range of $360^{\circ }$, which returns the coordinate information of all obstacles within the detection range of the sensor. We also assume that the real-time coordinate information of the USV is available with an on-board GPS sensor.

A sensed environment state of the USV is composed of three feature elements: the USV’s own coordinates, the perceived obstacle coordinate information organized as a vector, and the USV’s current distance from the destination. Then, the state vector of the USV, *s*, can be expressed by concatenating the aforementioned three vectors as follows:

(1) $$s={\left[{\left(x,y\right)}_{usv},M,d\right]}^{T},$$

where *M* represents the sensing information within the sensor’s perceptual range, and ${\left(x,y\right)}_{usv}$ is the real-time coordinates of the USV. The Euclidean distance between the USV and the destination is shown in (2):

(2) $$d=\sqrt{{\left(x-x_{goal}\right)}^{2}+{\left(y-y_{goal}\right)}^{2}}.$$

In  (2), $x_{usv}$, $y_{usv}$ represent the USV coordinates, and $x_{goal}$, $y_{goal}$ represent the destination coordinates. The environment sensing results regarding the obstacles are encoded into a binary image, where, following the rule of grid map generation, the white cells represent the available area and black cells indicate the position of obstacles.

### 3.2. Action Space

**Figure 4:**
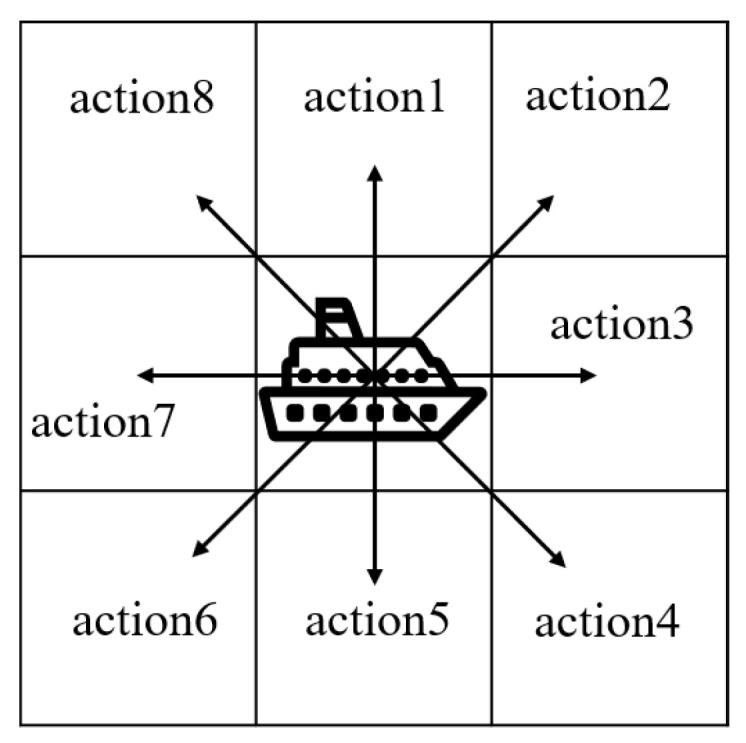
Actions of the USV.

We assume that the USV maintains a constant cruising speed measured in nautical miles per hour. Accordingly, we define the action set of the USV as consisting of eight discretized moving directions. Without loss of generality, we consider that, at each time slot, the USV can move from its current grid to any of the adjacent eight grids, as shown in Figure 4. In our algorithm, we use one-hot coding to represent the eight possible actions *a*.

### 3.3. Reward Function

We consider that our objective is to maximize the expected cumulative discounted reward along time for the USV. Therefore, the design of the instantaneous reward function is the key factor to determine the way of strategy adapation in the reinforcement learning process.

(3) $$r\left(s,a\right)=r_{1}\left(s,a\right)+r_{2}\left(s,a\right)+r_{3}\left(s,a\right)$$

where *s* and *a* are defined in Section 2.1 and Section 2.2, which represent state space and action space, respectively.

With our algorithm design, the instantaneous reward function consists of three parts: the reward for the USV’s distance from the destination $r_{1}$, the penalty for collision with obstacles $r_{2}$, and the one-time reward/penalty for arriving at the destination or moving out of bounds $r_{3}$.

We define $r_{1}$ as follows:

(4) $$\begin{array}{c} r_{1}\left(s,a\right)=\left\{\begin{array}{cc} 5 & \mathrm{if}d_{t}<d_{t-1},\\ -7 & \mathrm{if}d_{t}\geq d_{t-1}, \end{array}\right. \end{array}$$

where $d_{t}$ represents the distance (see (2)) from the destination at time slot *t*. (4) indicates that, when the USV is closer to the target position after taking a one-step action, the learning algorithm will give it an appropriate reward. Otherwise, a penalty will be imposed. Comparatively, $r_{2}$ gives the penalty for collisions with obstacles:

(5) $$\begin{array}{c} r_{2}\left(s,a\right)=\left\{\begin{array}{cc} -5 & \mathrm{if}I({\left(x,y\right)}_{usv}\left(s,a\right),\mathrm{ob}),\\ -0.002 & \mathrm{if}otherwise, \end{array}\right. \end{array}$$

where $I({\left(x,y\right)}_{usv},\mathrm{ob})$ is the indicator function showing whether the designated USV position collides with an obstacle. Notice that we introduce a small penalty for the situation of the USV remaining idle on the map. This method encourages the USV to reach its destination as soon as possible.

Finally, $r_{3}\left(s,a\right)$ indicates that the USV is either rewarded upon successful completion when it reaches the target location or incurs a failure penalty if its coordinates exceed the designated boundary or if collisions occur:

(6) $$\begin{array}{c} r_{3}\left(s,a\right)=\left\{\begin{array}{c} 100,\;\;\mathrm{if}d_{t}\leq 0.1,\\ -5,\;\;\;\mathrm{if}I\left({\left(x,y\right)}_{usv}\left(s,a\right),\mathrm{ob}\right)\mathrm{or}I\left({\left(x,y\right)}_{usv}\left(s,a\right),\mathrm{out}-b\right), \end{array}\right. \end{array}$$

where $I({\left(x,y\right)}_{usv},\mathrm{out}-b)$ indicates whether the USV is out of the map boundary. With (5) and (6), when the USV reaches the target position, a large one-time reward is given. When the USV is out of boundary or collides with an obstacle, a penalty of −5 is given at task termination.

### 3.4. Improved PPO with Better Generalization Capability

In this section, we propose to adapt the PPO algorithm in two stages to solve the path planning problem in USV.

#### 3.4.1. Neural Network Design with Convolutional Layers

We employ convolutional layers to extract the feature of the obstacle map obtained from the sensing results. The calculation of the *i*-th feature graph of the *n*-th convolutional layer is defined as follows:

(7) $$x_{i}^{n}=f\left(0,\sum_{j\in M_{i}}x_{j}^{n-1}k_{ji}^{n}+b_{i}^{n}\right),$$

where $M_{i}$ is the set of feature graphs, for which the initial input is given in (7), $k_{ji}^{n}$ is the *i*-th convolution kernel of the *n* layer, $b_{i}^{n}$ is the *i*-th configuration of the *n* layer, and f(·) is the activation function, for which we use the ReLU function in our designed neural network. For our adopted PPO neural network, the details of the network parameters are given in Table 1.

#### 3.4.2. PPO Algorithm

Proposed by OpenAI [30], the PPO algorithm introduces a new objective function that can be updated in small batches with multiple training steps. This solves the problem where the step size is difficult to determine as in the vanilla Policy Gradient (PG) algorithm. As one PG algorithm, the main idea of PPO is to use gradients to update the USV’s strategy π(a|s) in order to maximize the expected cumulative reward. In a PG algorithm, the objective function of the network parameter θ update is as follows:

(8) $$L^{PG}\left(\theta \right)=E_{t}\left[\log\pi_{\theta }\left(a_{t}\left|s_{t}\right.\right)A_{t}\right],$$

where $A_{t}$ is the advantage function estimating the value of taking action $a_{t}$ in state $s_{t}$ compared to the average expected return. $A_{t}$ will be given in our later discussion.

The PG algorithm is highly sensitive to the step size, making it challenging to select an appropriate value. To overcome this limitation, PPO uses the ratio of the action probability $\pi_{\theta }\left(a_{t}\left|s_{t}\right.\right)$ under the current strategy to the action probability $\pi_{\theta_{old}}\left(a_{t}\left|s_{t}\right.\right)$ of the previous strategy to observe the effect of the agent’s action. The ratio of old and new strategies is recorded as

(9) $$r_{t}\left(\theta \right)=\frac{\pi_{\theta }\left(a_{t}\left|s_{t}\right.\right)}{\pi_{\theta_{old}}\left(a_{t}\left|s_{t}\right.\right)}.$$

If the ratio between the new and old strategy functions is $r_{t}\left(\theta \right)>1$, it indicates that the probability of the action occurring under this policy is higher than that of the previous policy. Otherwise, the probability is lower than the previous policy. The objective function used for training is given as follows:

(10) $$L^{CPI}\left(\theta \right)=E_{t}\left[\frac{\pi_{\theta }\left(a_{t}\left|s_{t}\right.\right)}{\pi_{\theta_{old}}\left(a_{t}\left|s_{t}\right.\right)}A_{t}\right]=E_{t}\left[r_{t}\left(\theta \right)A_{t}\right],$$

where $A_{t}$ is the advantage function:

(11) $$A_{t}=Q(s_{t},a_{t})-V(s_{t},a_{t})$$

In (11), $Q(s_{t},a_{t})$ is the cumulative reward value of taking action $a_{t}$ under state $s_{t}$, and $V(s_{t},a_{t})$ is the estimated state value. $A_{t}>0$ indicates that the current action is better than the average action and the learning process increases the probability of choosing the action. Otherwise, when $A_{t}<0$, it means that the action is worse than the average action and the probability of choosing the action will be reduced.

**Figure 5:**
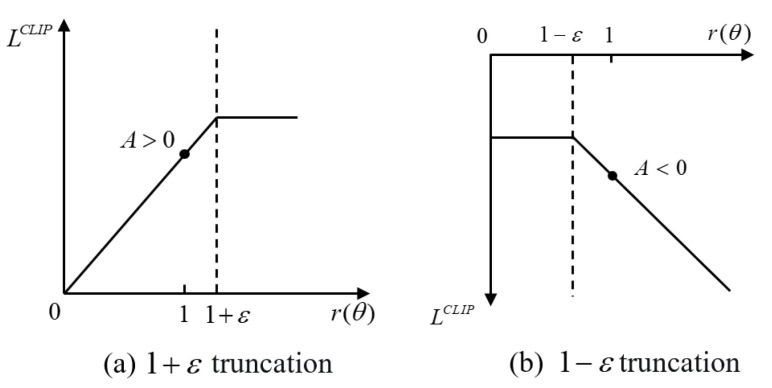
Restricted range of objective function.

The PPO algorithm improves the stability of training agent behaviors in PG by constraining policy updates to a small range. The objective function of PPO is improved from (10) as

(12) $$L^{CLIP}\left(\theta \right)=E_{t}\left[\min\left(r_{t}\left(\theta \right)A_{t},CLIP\left(r_{t}\left(\theta \right),1-\varepsilon ,1+\varepsilon \right)A_{t}\right)\right],$$

where the clip function is a truncation function that limits the value of the old and new policy parameters r(θ) to the interval [1−ε,1+ε]. ε is a truncation constant used to assist in setting the range of policy updates; it is usually set to 0.1 or 0.2. Figure 5a,b illustrate these two situations of truncation [30], respectively. The pseudo code for the PPO–Clip algorithm is given in Algorithm 1.

**Algorithm 1**: Pseudo code of the PPO–Clip algorithm
1:Input: initial policy parameters $\theta_{0}$, value function parameters $\phi_{0}$, clipping threshold ϵ.2:**for** k=0,1,2,…do3:  Collect set of trajectories $D_{k}=\tau_{i}$ by running policy $\pi_{k}=\pi \left(\theta_{k}\right)$.4:  Compute advantage estimates, $A_{t}^{\pi_{k}}$ (using any method of advantage estimation)  based on the current value function $V_{\phi k}$.5:  Update the policy by maximizing the PPO–Clip objective:
$\theta_{k+1}=\arg\max_{\theta }L_{\theta_{k}}^{CLIP}\left(\theta \right)$  typically via stochastic gradient ascent with Adam, where
$L_{\theta_{k}}^{CLIP}\left(\theta \right)=\underset{\tau ∼\pi_{k}}{E}\left[\sum_{t=0}^{T}\left[\min(r_{t}\left(\theta \right)A_{t}^{\pi_{k}},clip\left(r_{t}\left(\theta \right),1-\epsilon ,1+\epsilon \right)A_{t}^{\pi_{k}})\right]\right]$6:  Fit value function by regression on mean-squared error:
$\phi_{k+1}=\arg\min_{\phi }\frac{1}{\left|D_{k}\right|T}\sum_{\tau \in D_{k}}\sum_{t=0}^{T}{\left(V_{\phi }\left(s_{t}\right)-R_{t}\right)}^{2}$  typically via some gradient descent algorithm.7:
**end for**



## 4. Experiments

In this section, we provide a series of numerical simulation results to evaluate the performance of our proposed algorithm. For marine environment simulation and USV strategy training, all the experiments are performed with Pytorch 2.0.1 on a desktop machine with 128 G memory and hardware acceleration using a GeForce RTX 3090Ti GPU from NVIDIA Santa Clara, CA, USA. We aim to validate the generalizability of the proposed algorithm by modifying three conditions: endpoint coordinates, map, and the number of training sets. We conduct simulation experiments from various aspects to verify the effectiveness of our approach. Experiment 1 focuses on a USV obstacle avoidance simulation using the algorithm proposed in this paper. In Experiment 2, we test the generalization capability of the proposed algorithm by changing the endpoint. Similarly, in Experiment 3, we explore the algorithm’s generalization under different sea maps. Experiment 4 involves training an additional network model by increasing the number of maps used for training. This model is then used to assess the generalization of the proposed algorithm in the simulation environment of Experiment 2. Finally, Experiment 5 involves comparing the performance of the proposed algorithm with other algorithms, thereby demonstrating its effectiveness.

### 4.1. Generalization Definition and Modeling

For reinforcement learning (RL), the generalization ability refers to when the reinforcement learning model is trained in the training environment and the performance of the model is verified in the test environment for the same task in the same domain. In supervised learning, some predictor is trained on a training dataset, and the performance of the model is measured on a held out testing dataset. It is often assumed that the data points in both the training and testing dataset are drawn independently and identically distributed from the same underlying distribution. The generalization gap in supervised learning for a model ϕ with training and testing data $D_{train}$, $D_{test}$ and loss function *L* is defined as

(13) $$GenGap\left(\phi \right):=E_{\left(x,y\right)∼D_{test}}\left[L\left(\phi ,x,y\right)\right]-E_{\left(x,y\right)∼D_{train}}\left[L\left(\phi ,x,y\right)\right].$$

This gap is used as a measure of generalization, specifically, a smaller gap means a model generalizes better. Generalization refers to a class of problems, rather than a specific problem. Thus, the generalization measure in RL is shown in Equation (13).

To discuss generalization, we need a way of talking about a collection of tasks, environments, or levels; the need for generalization emerges from the fact that we train and test the policy on different collections of tasks. To formalize the notion of a collection of tasks, we start with the Contextual Markov Decision Process (CMDP). The CMDP is shown as Equation (14):

(14) $$M=(S^{\prime },A,O,R,T,C,\phi ,p\left(S^{\prime }\left|C\right.\right),p\left(C\right)).$$

where $S^{\prime }$ is the underlying state space; *A* is the action space; *O* is the observation space; *R* is the scalar reward function; *T* is the Markovian transition function; *C* is the context space; and $\phi :S^{\prime }\times C\to O$ is the emission or observation function. We factorize the initial state distribution as shown in (15):

(15) $$p\left(S^{\prime },C\right)=p\left(C\right)p\left(S^{\prime }\left|C\right.\right).$$

and we call p(C) the context distribution. This distribution is what is used to determine the training and testing collections of levels, tasks, or environments. We now describe the class of generalization problems we focus on, using the CMDP formalism.

**Figure 6:**
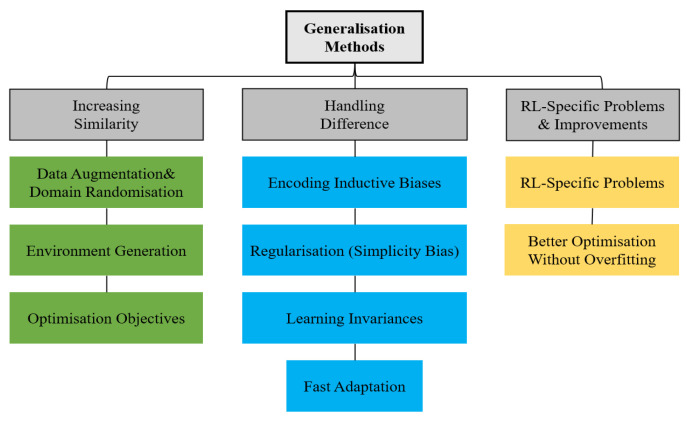
Categorization of methods for tackling generalization in reinforcement learning [31].

All else being equal, the more similar the training and testing environments are, the smaller the generalization gap and the higher the test time performance. The categorization of the methods for tackling generalization in RL is shown in Figure 6.

In this paper, the generalization of the model is verified by holdout validation and data augmentation. For the holdout validation method, the map set is divided into the training set and the test set. The training set specifically refers to the simulation data in Experiment 1. The test set refers to the simulation data in Experiment 2 and Experiment 3. The results of Experiment 2 and Experiment 3 show that the USV can successfully reach the end point under different test sets. For the data augmentation method, by transforming and expanding the training data, more samples are introduced. Then, Experiment 4 tests the generalization of the model under the condition of multiple training data sets by using the simulation environment of Experiment 2.

### 4.2. Simulation Experiment

#### 4.2.1. Experimental Platform Description and Training Parameters

**Figure 7:**
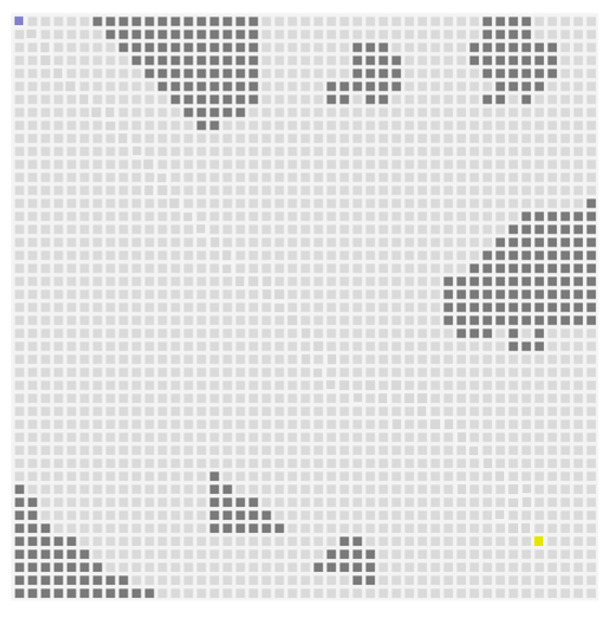
USV simulation in the sea environment.

The USV simulation environment in this paper is shown in Figure 7, which is extracted from the data as illustrated in Figure 1. In this environment, the USV is represented by the blue square in the figure. The gray squares represent obstacles. The yellow squares represent the end of the path. USV continuously learns and explores its strategy, following the method proposed in this paper. The entire scene is reset at the end of each round or when the maximum number of time steps is exceeded in a single round. The detection radius of the radar is set to 153 m. In the simulation experiment, the performance indicator values include the iterations used by the USV to reach the end point, the time needed for algorithm convergence, and the average reward obtained. Among them, the step numbers can be reflected by the path diagram of each experiment. The allowable number of the training time steps in this experiment is $3\times 10^{5}$, and the PPO parameter settings are shown in Table 2. The convergence time and average reward are shown in Table 3.

#### 4.2.2. Experimental Results and Analysis

**Figure 8:**
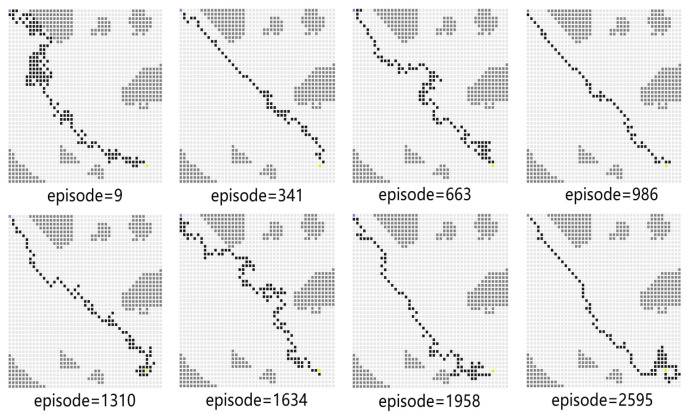
The planned path for Experiment 1.

**Figure 9:**
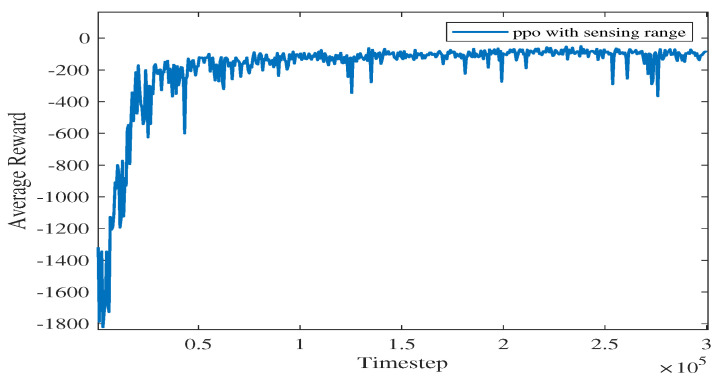
The average reward evolution for Experiment 1.

For Experiment 1, the path trace following the proposed algorithm and the convergence tendency of the average reward are shown in Figure 8 and Figure 9, respectively. We also record the snapshot of the paths for the USV to reach the destination in each episode, as shown in Figure 8. The average reward converges when the episode reaches 341. During the training process, with the improvement of the strategy, the number of steps is reduced when the USV reaches the end at each episode. To find a better strategy, the PPO algorithm explores the unknown action space. Therefore, when the episode reaches 1634, the number of steps is increased.

As shown in Figure 9, as the iteration time steps increase, the average reward converges when the algorithm iterates to roughly $10^{5}$ time steps. The final converged reward fluctuates between −111.34 and −125.55, which shows that the proposed algorithm is basically converging.

**Figure 10:**
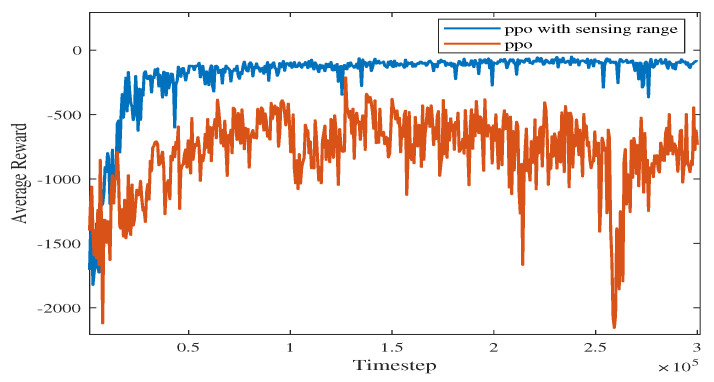
Comparison of training processes with different methods using PPO.

In Figure 10, the vanilla PPO algorithm with zero sensing range is used for path planning under the same environment. It can be seen from the figure that PPO using convolutional layers achieves a better cumulative reward. In the absence of a sensing range, the USV is not able to handle different obstacle environments (see the near-to-end stage of the training process). The experimental results show that increasing the sensing range can greatly improve the convergence efficiency of USV as well as the obstacle-handling capability.

**Figure 11:**
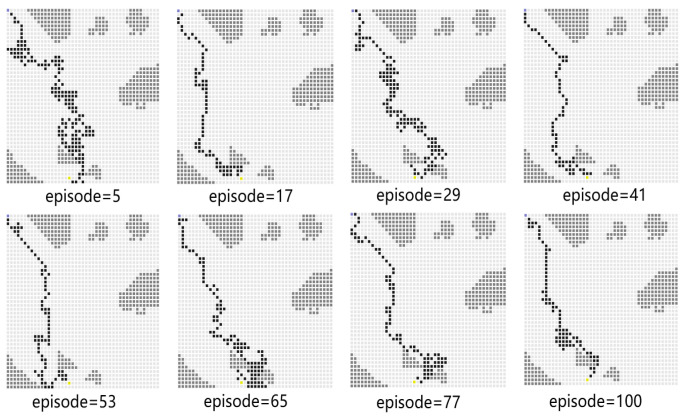
The path diagram on the testing map for Experiment 2.

In Experiment 2, the generalization capability of the proposed algorithm is verified by modifying the path endings. The starting point coordinates of the simulation environment remain unchanged, and the end point coordinates are changed from (40, 40) to (19, 44). The path diagram of the test process is shown in Figure 11, which shows that the USV successfully reaches the end point. During the test process, the PPO algorithm will explore the unknown action space to find a better strategy. When the episode reaches 77, the average reward value decreases sharply, and the number of steps is increased. At the 100th episode, the USV uses the least number of steps to reach the end point and receives the highest average reward. After changing the end point of the simulation environment to more complex areas, the models trained by the proposed algorithm can also guide USV to reach the end point. It indicates that the proposed algorithm has a strong generalization capability.

**Figure 12:**
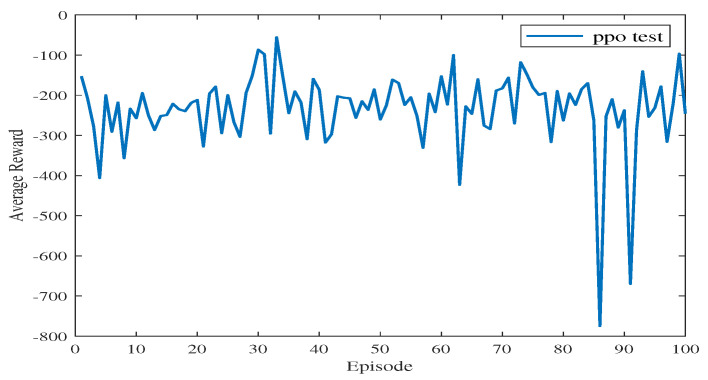
The average reward during testing in Experiment 2.

Figure 12 shows the average reward of the proposed algorithm with sensing capability after changing the end point of the simulation environment. The total training session is 100 episodes. As shown in Figure 12, the blue line takes a few time steps to reach the average reward convergence value, and the average reward for testing is stable at −233.84. This means that the USV can find a safe and collision-free path in the simulation environment after changing the end point of the path planning.

**Figure 13:**
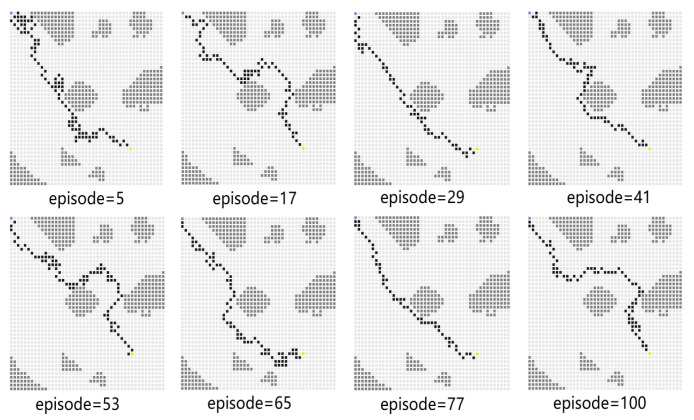
The path diagram on the testing map for Experiment 3.

**Figure 14:**
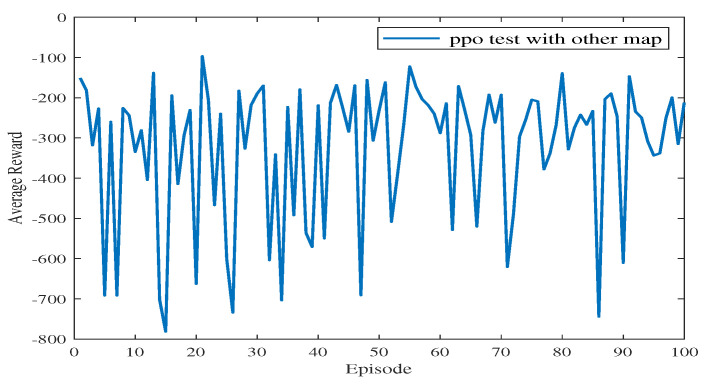
The average reward during testing in Experiment 3.

In Experiment 3, the generalization of the proposed algorithm is further verified by modifying the simulation map. In Experiment 3, the training map is changed to a new one, including the obstacles on the map and the end point coordinates. The path planning diagram for the test is shown in Figure 13. The algorithm proposed in this paper can make the USV bypass from above or below the obstacle and successfully reach the end point. At the 77th episode, the USV takes the least number of steps to reach the end point and receives the highest average reward. As shown in Figure 14, the blue line represents the average reward convergence diagram after 100 episodes of testing. The average reward is −323.18.

**Figure 15:**
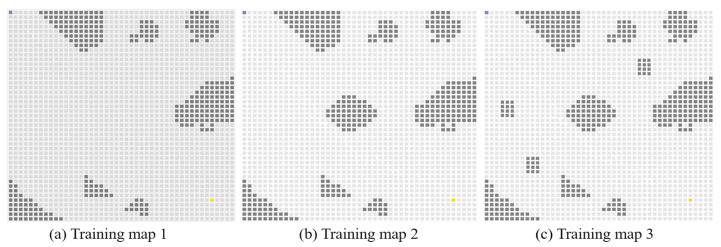
Illustration of expanded training sets for generalization.

**Figure 16:**
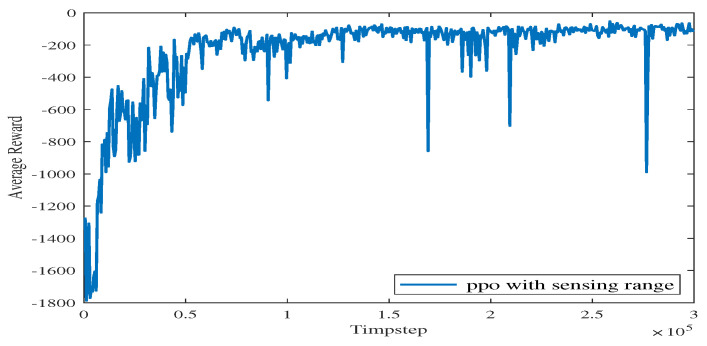
The average reward in Experiment 4.

In Experiment 4, we increase the size of the training map sets, and parts of the maps are shown in Figure 15. As shown in Figure 16, the blue line represents the average reward convergence curve obtained by training with the three maps. The final average reward fluctuates between −100.28 and −168.54, indicating that the proposed algorithm basically tends to converge.

**Figure 17:**
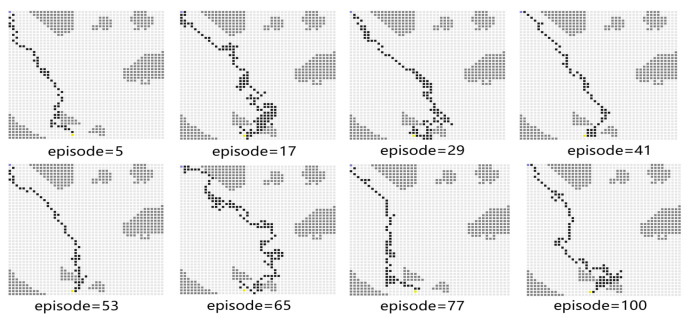
The path snapshots obtained during testing in Experiment 4.

Figure 17 shows the path diagram in testing after expanding the training set. In some of the rounds shown therein, the USV successfully reaches the end point, which demonstrates the generalization capability of the algorithm proposed in this paper.

**Figure 18:**
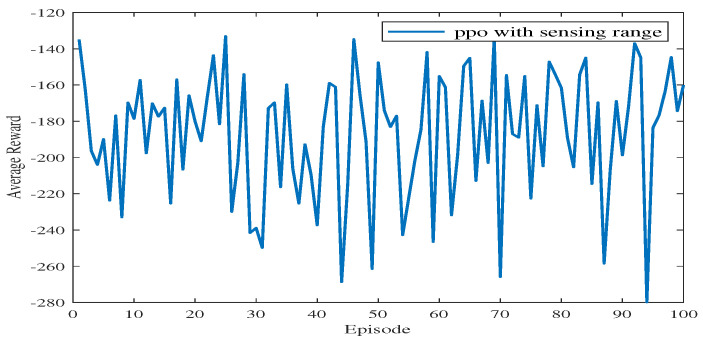
The average rewards during testing in Experiment 4.

**Figure 19:**
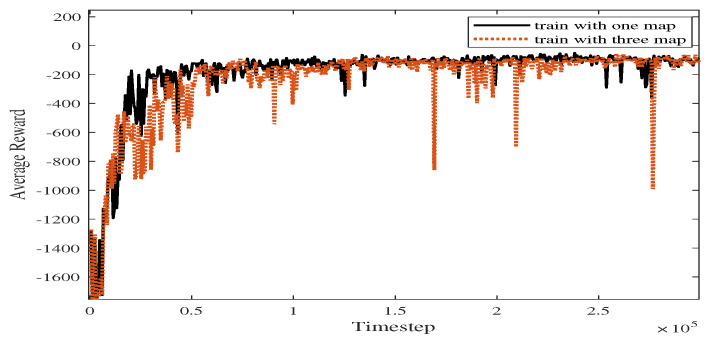
Comparison of average reward curves in Experiments 1 and 4.

As shown in Figure 18, the blue line represents the average reward convergence diagram after 100 rounds of testing. The average reward is −186.84. After changing the training atlas, the average reward convergence curve obtained is shown in Figure 19, where the solid black line represents the average reward convergence graph after training on a static graph, and the red dotted line represents the average reward convergence graph after training with three static maps.

#### 4.2.3. Comparative Experiment

**Figure 20:**
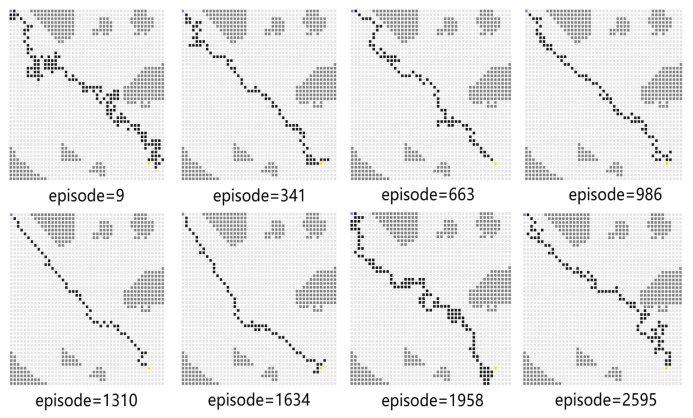
PPO-based path planning diagram.

**Figure 21:**
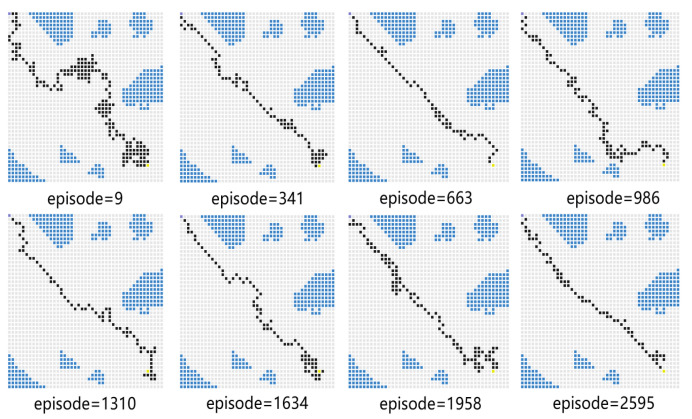
Path diagram with SAC.

**Figure 22:**
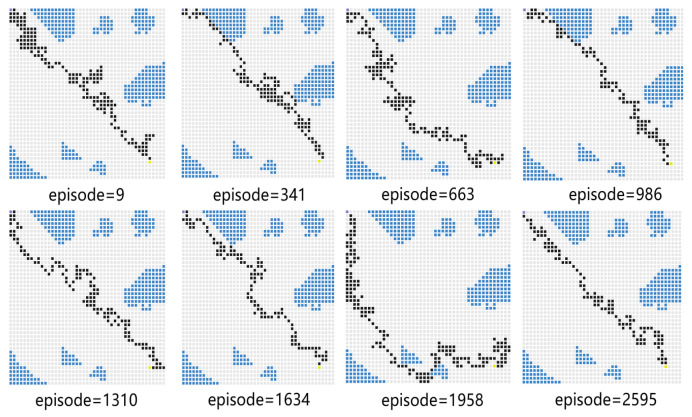
Path planning diagram with DQN.

Experiment 5 verifies the effectiveness of the proposed algorithm by comparing its performance with several baseline algorithms. For comparison, the USV performs obstacle avoidance tasks based on the SAC algorithm, the PPO algorithm, the DQN algorithm, and the proposed PPO algorithm with a sensing range. We compare the average reward obtained by these algorithms in the same scenario and the convergence time steps taken to reach the target position. The snapshots of different algorithm results are given in Figure 20, Figure 21 and Figure 22. In the process of training, with the improvement of strategy, the USV becomes more and more certain and the average reward value is convergent. As shown in Figure 20, when the episode reaches 1958, the PPO algorithm explores a new action space, and the number of steps is increased. In Figure 21, at the 1634th episode, the USV falls into a local optimum near the end point, and the number of steps is increased. As shown in Figure 22, the number of steps for the USV to reach the end of each round gradually decreases, and the final average reward is convergent.

**Figure 23:**
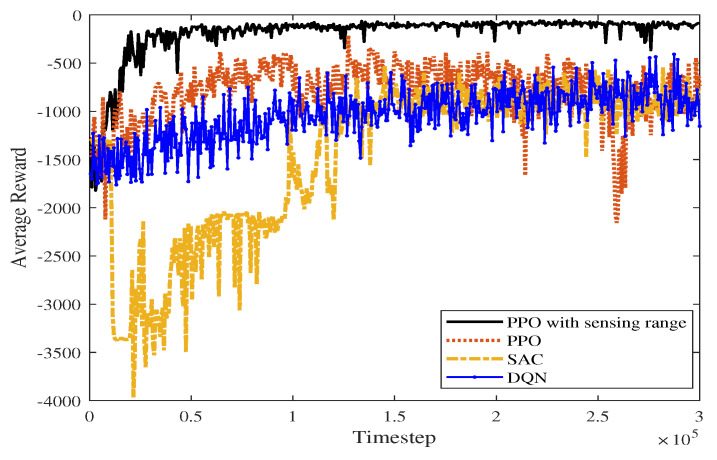
Average reward comparison graph of four algorithms in a static scene.

Figure 23 shows the average reward curve of the four algorithms as the time steps increase in a static environment. The algorithm proposed in this paper has less fluctuation in the early training process than the PPO, DQN, and SAC algorithms. As shown in Table 3, the proposed algorithm, PPO algorithm, DQN algorithm, and SAC algorithm converge in 20,400, 59,400, 136,800 and 142,000 time steps, respectively. According to Figure 23, SAC fluctuates greatly before the average reward converges. The proposed algorithm takes advantage of the improved perceptual capability of PPO and accumulates higher rewards. In the late stages of training, the average reward converges to around −117.574.

## 5. Conclusions and Future Work

In this paper, the path planning algorithm based on improved PPO for USVs is proposed. The convolutional layers are adapted as the state-feature extraction module for handling the sensing data in the sea environment. Such design helps the USV in making proper decisions when facing unknown environments, since the obstacle patterns have been learned with the CNN layers. Simulation experiments show that the average reward and convergence rate of the proposed algorithm are significantly better than the other baselines algorithms, which lays a foundation for subsequent research.

In our future work, given that a 2D raster model has a limitation in reflecting the influence of waves and winds at sea, we will expand our sea model to a 3D ocean area. In particular, we will focus on the stochastic influence of the environment on the USV strategies, especially in terms of motion control of the USV.

## Figures and Tables

**Table 1:** Parameters of the actor–critic networks for PPO with convolution layers.

Model Network	Input	Kernel-Size	Fc	Activation Function	Output
Actor	state-dim	3×3,1,1	16×16×45	Relu	action-dim
Critic	state-dim	3×3,1,1	16×16×45	Relu	status-value

**Table 2:** Hyperparameters for the proposed PPO algorithm.

Parameters	Value
Actor learning rate	$1\times 10^{-4}$
Critic learning rate	$3\times 10^{-4}$
Discount factor	0.99
Batch size	1500
Time steps	$3\times 10^{5}$
Clip range	0.2

**Table 3:** Comparison of experimental data in a static environment.

Comparative Experiment	Average Reward	Convergence Time Steps $\left(10^{4}\right)$
PPO with sensing range	−117.574	2.04
PPO	−535.18	5.94
DQN	−705.675	13.68
SAC	−840.58	14.2

## Data Availability

All data generated or analyzed during the study are included in this published article.
